# Effects of trait empathy and expectation on the processing of observed actions

**DOI:** 10.3758/s13415-020-00857-7

**Published:** 2020-12-09

**Authors:** Christine Albrecht, Christian Bellebaum

**Affiliations:** Institute of Experimental Psychology, Heinrich Heine University Düsseldorf, Universitätsstraße 1, 40225 Düsseldorf, Germany

**Keywords:** Action observation, Expectation, Empathy, ACC, Error processing

## Abstract

Recent evidence suggests that the processing of observed actions may reflect an action prediction error, with more pronounced mediofrontal negative event-related potentials (ERPs) for unexpected actions. This evidence comes from an application of a false-belief task, where unexpected correct responses elicited high ERP amplitudes. An alternative interpretation is that the ERP component reflects vicarious error processing, as objectively correct responses were errors from the observed person’s perspective. In this study, we aimed to disentangle the two possibilities by adding the factor task difficulty, which varied expectations without affecting the definition of (vicarious) errors, and to explore the role of empathy in action observation. We found that the relationship between empathy and event-related potentials (ERPs) mirrored the relationship between empathy and behavioral expectancy measures. Only in the easy task condition did higher empathy lead to stronger expectancy of correct responses in the true-belief and of errors in the false-belief condition. A compatible pattern was found for an early ERP component (150–200 ms) after the observed response, with a larger negativity for error than correct responses in the true-belief and the reverse pattern in the false-belief condition, but only in highly empathic participants. We conclude that empathy facilitates the formation of expectations regarding the actions of others. These expectations then modulate the processing of observed actions, as indicated by the ERPs in the present study.

**Supplementary Information:**

The online version contains supplementary material available at 10.3758/s13415-020-00857-7.

Monitoring one’s actions plays an important role in goal-directed behavior, making it possible to adapt performance quickly when necessary. An important aspect of this is the recognition of committed errors. For example, when you open the top drawer in the kitchen looking for a spoon, although you know that spoons are in the bottom drawer. In this case, you usually notice your error immediately. The neural processing of own errors has been thoroughly investigated over the past 30 years. In the 1990s, researchers first described a negative deflection in the event-related potentials (ERPs) of electroencephalography (EEG) data after error commission (Falkenstein, Hohnsbein, Hoormann & Blanke, 1991). This component, peaking within 100 ms after error commission, is called error negativity (Ne) or error-related negativity (ERN; Falkenstein et al. 1991; Falkenstein, Hoormann, Christ & Hohnsbein, 2000; Gehring, Goss, Coles, Meyer & Donchin, 1993; see also Gehring, Liu, Orr & Carp, 2012; Holroyd & Coles, 2002).

Error monitoring, however, is not limited to own errors. A negative deflection in ERPs similar to the ERN has been demonstrated for study participants observing others’ errors. This deflection is referred to as observer error-related negativity (oERN; van Schie, Mars, Coles & Bekkering, 2004; see also Koban & Pourtois, 2014). As the ERN (Falkenstein et al., 2000; Dehaene, Posner & Tucker, 1994; Ridderinkhof, Ullsperger, Crone & Nieuwenhuis, 2004; Taylor, Stern & Gehring, 2007; see also Gehring et al., 2012), the oERN displays a frontocentral topography and is believed to originate from the anterior cingulate cortex (ACC) (Miltner, Brauer, Hecht, Trippe, & Coles, 2004; van Schie et al., 2004; see also Koban & Pourtois, 2014). Recent findings also indicate the involvement of other brain regions. Ullsperger, Danielmeier, & Jocham (2014) suggest the posterior medial frontal cortex (pMFC) as a generator of performance monitoring components, including the anterior and posterior midcingulate cortex, as well as presupplementary and supplementary motor areas and the posterior dorsomedial prefrontal cortex. For action observation specifically, the superior temporal sulcus might additionally contribute to oERN generation (Ninomiya, Noritake, Ullsperger, & Isoda, 2018). In comparison to the ERN, the amplitude of the oERN is smaller (van Schie et al., 2004; Miltner et al., 2004). Not surprisingly, it also peaks later relative to the eliciting event (see Gehring et al., 2012), because it is not time-locked to a self-performed response but to an observed action. Moreover, its latency seems to vary between 130 and 300 ms, depending on the experimental paradigm (Bates, Patel, & Liddle, 2005; Koban, Pourtois, Vocat, & Vuilleumier, 2010 as opposed to Carp, Halenar, Quandt, Sklar & Compton, 2009; de Bruijn & von Rhein, 2012; Miltner et al., 2004; van Schie et al., 2004). The monitoring of others’ actions can be considered a social process. For example, it is of particular importance for joint actions, when own actions are synchronized with others’ (Loehr, Kourtis, Vesper, Sebanz, & Knoblich, 2013; Moreau, Candidi, Era, Tieri, Aglioti, 2020).

In recent years, the understanding of how performance monitoring is represented in the human brain and of the processes that underlie the ERN and related ERP components has changed. Increasing evidence supports the assumption that unexpected events, rather than errors, mainly drive ERP components and brain activity previously associated with error commission or error feedback for self-performed actions (Alexander & Brown, 2011; Ferdinand, Mecklinger, Kray, & Gehring, 2012; Wessel, Danielmeier, Morton, & Ullsperger, 2012). As accuracy and expectancy are usually confounded, at least for easy tasks, in which errors are rare, it cannot be differentiated whether an ERP component reflects an error or an unexpected event. This further applies to the ERPs associated with observed errors: Do they actually reflect the accuracy or the expectancy of these actions or both? It is assumed that when other people’s actions are observed, predictions are formed that are then compared to the actually performed actions (i.e. the outcome of the prediction). If the two do not match, an action prediction error occurs (Burke, Tobler, Baddeley, & Schultz, 2010; Donnarumma, Costantini, Ambrosini, Friston & Pezzulo, 2017; Flanagan & Johansson, 2003), which is independent of the valence of the response, i.e., equally pronounced for an unexpected error and for an unexpected correct action.

An expectancy effect on a mediofrontal ERP component for observed actions has indeed been demonstrated in a previous study by our group. In that study, we applied a paradigm in which participants observed a person playing a stimulus-response task, the two-shell-game (see *Methods* for details). In this game, participants have to track under which of two shells a ball is hidden. Because this task is quite easy, erroneous responses by the observed person were unexpected (Kobza & Bellebaum, 2013). The task, however, also entails a false-belief condition: in this, observers had exclusive access to task-related knowledge that made correct responses unexpected. In this condition, the mediofrontal ERP component showed larger negative amplitudes after (unexpected) correct than (expected) error responses.

This finding appears to support the assumption that negative medio-frontal ERPs reflect that something unexpected happens. However, there may be an alternative interpretation. In the task we applied, as in real life, the observed person’s subjective error could differ from the actual, objective error. To return to the introductory example: When you know that the spoons have been moved to the top drawer, but the observed person does not, then opening the top drawer looking for a spoon is objectively correct, but an error from the observed person’s point of view. Objective and subjective error are dissociated in a false-belief condition, but not in the true-belief condition. Thus, the mediofrontal ERP component may also code vicarious error processing: Both conditions for which higher amplitudes were found, (objective) errors in the true-belief condition and (objectively) correct actions in the false-belief condition, are subjective errors to the naïve observed person. This interpretation in terms of vicarious error processing appears to be supported by a recent study where we found that trait empathy, measured by the empathy quotient (EQ) (Baron-Cohen & Wheelwright, 2004), was related to the processing of those actions in the two-shell-game that represented errors from the observed person’s perspective (Bellebaum, Ghio, Wollmer, Weismüller, & Thoma, 2020). In participants with higher empathy scores, particularly large amplitudes of the mediofrontal negative ERP component were found in these conditions. This finding, however, was interpreted in terms of a facilitatory effect of empathy on the generation of expectations regarding observed actions. To summarize, it is not clear what cognitive process is primarily reflected in ERPs following observed actions—that is, whether they represent (subjective) accuracy from the perspective of the observed person and thus vicarious error processing or the (un)expectedness of the observed action, nor what role empathy plays in this respect. Although it shares some features with the oERN as described in the literature (van Schie et al., 2004), we will refer to the ERP component(s) of interest as negative mediofrontal ERP component in order to leave its functional significance undetermined.

In the present study, we aimed to disentangle effects of vicarious errors and action expectancy on the processing of observed actions by adding the factor task difficulty, because it should affect the latter but not the former. The two-shell game described above (Kobza & Bellebaum, 2013) can be considered an easy task (low level of difficulty), yielding clear expectations regarding the upcoming response in terms of accuracy, with correct responses being expected in the true-belief condition and errors in the false-belief condition. We reasoned that in a task of high difficulty, expectations would not be as clear. As there were only two response options, observers should expect that the observed person guesses more often, so that expectations concerning accuracy of the observed response would be nearer to chance level. In the introductory example, increasing task difficulty could correspond to looking for the drawer with the spoons in an unfamiliar kitchen, where you can more or less only rely on guessing. Comparing ERPs elicited by responses in high and low difficulty trials allows us to disentangle effects of action expectancy and vicarious error processing. The correct and incorrect answers remain the same for both high and low difficulty trials—subjectively, from the observed player’s point of view, and also objectively. If the mediofrontal ERP component reflects vicarious error processing, no effect of task difficulty would thus be expected. However, if the observers’ expectancy of the observed action determines its processing, task difficulty should have an effect. This notion is only true, however, if task difficulty indeed affects the expectancy of the observed response.

We hypothesized that the amplitude of the mediofrontal ERP component in response to observed actions that we and others described before (Bellebaum et al., 2020; Bates et al., 2005; Kobza & Bellebaum, 2013; van Schie et al., 2004) primarily reflects the expectancy of the observed responses rather than vicarious errors. In addition, we aimed to clarify the role of trait empathy in the processing of observed responses. By adding the factor task difficulty, we aimed to create more variance concerning the expected accuracy of the observed action, so that not only effects of expectancy and vicarious errors could be dissociated, but also the relationship between empathy and expectation effects regarding observed error monitoring could be examined.

## Methods

### Participants

A total of 38 participants took part in the study. As Mixed Linear Models are not yet used comprehensively and methods for power calculations have only emerged in the last years and require effect sizes for specific effects and interactions (Green & MacLeod, 2016), we chose this sample size based on studies using correlations to investigate the effect of continuous measures of trait empathy on action monitoring (Lockwood, Apps, Roiser, & Viding, 2015; Newman-Norlund, Ganesh, van Schie, de Bruijn, & Bekkering, 2009; Shane, Stevens, Harenski, & Kiehl, 2009). In these studies, sample sizes were between 20 and 31 participants. Five of the acquired participants were excluded from data analyses, either due to technical problems (four) or because the dependent variables derived from the EEG data were outliers in the analysis (one, see below for details). The remaining 33 participants (12 men) were between 18 and 33 years old (*M* = 22.8, *SD* = 3.6). They reported no previous or existing psychiatric or neurological illnesses and took no regular medication that could affect the nervous system. All participants had normal or corrected-to-normal vision and were German native speakers. Participants received course credit for taking part in the experiment. The study was approved by the ethics committee of the Faculty of Mathematics and Natural Sciences at Heinrich Heine University Düsseldorf, Germany.

### Experimental Task

The paradigm in this study was an adaptation of the two-shell game used by Kobza and Bellebaum (2013) and Bellebaum et al. (2020). Participants were asked to observe another person as he played the game. Unbeknownst to the participants, the player was fictitious and the displayed trials were simulated. The (fictitious) male player was introduced with a name and a photo, in order to give the impression that the participants observed the performance of a real person. The game started with a ball being hidden under one of two shells. After multiple rotations of the two shells (2, 3, or 4 rotations, randomly determined), the fictitious player pointed a joystick towards the shell where he believed the ball to be hidden. The observers saw the game from above, which also meant that they could see the ball at any time during the trial and therefore knew immediately whether the observed player was right or wrong when he moved the joystick at the end of the trial. The player’s responses were balanced: half were correct responses (pointing to the shell covering the ball), and the other half were errors (pointing to the empty shell).

We aimed to modulate the observer’s expectations concerning the player’s responses by two factors. First, as in Kobza and Bellebaum (2013), a false-belief condition was introduced. That meant that the player was tricked in half of the trials (factor Trial Type): the ball was swapped between the two shells during one of the rotations. Observer participants were told that this was almost never visible to the player, while it was clearly visible to the observers themselves. If the player was tricked, the observers should expect a wrong rather than a correct answer of the player, because they believed that the player could not have seen the trick, and he would therefore assume that the ball was under the wrong shell. In the no-trick condition, respectively, the observers should expect the player to answer correctly.

As correct responses in the trick condition were errors from the perspective of the player, we added the factor Difficulty to the task, which aimed to disentangle vicarious errors and expectancy: the difficulty of keeping track of the ball was high in half of the trials, in that the shells were rotated more than three times faster (255 ms per rotation) than in the previous version of the experiment (850 ms per rotation), which was now considered the “slow” and thus low difficulty condition. Participants were told that due to the speed, it would be more difficult for the player to follow the shells with his eyes, so he had to guess more often in his decision under which shell the ball was. We assumed therefore that in low difficulty trials, observer participants would have stronger expectations regarding the player’s response accuracy than in the high difficulty condition, for which the expected accuracy would only be slightly higher or lower than chance level (i.e., 50%) in the no-trick and trick conditions, respectively.

The experiment was arranged in four blocks of 117 trials between which participants could take short breaks. In contrast to the procedure in our previous studies applying this paradigm (Kobza and Bellebaum, 2013; Bellebaum et al., 2020), trick and no-trick conditions were alternated between blocks, because otherwise the build-up of expectations concerning response accuracy by the observed person might have been too complex given that we introduced an additional factor. In two of these blocks, the player was always tricked, in the other two blocks, he was never tricked. Observers knew in advance that the next block would only contain trick or no-trick trials. The order of the blocks was balanced between participants; either the first two blocks were trick-trials and the last two no-trick trials, or vice versa. Before the first block was started, participants completed 12 practice trials (6 trick and 6 no-trick trials).

Half of the 234 trials of each Trial Type were high difficulty trials, the other half were low difficulty trials. The low difficulty and high difficulty trials were presented in random order in the two blocks of each of the two levels of the Trial Type factor (trick and no-trick). In half of the trials, the fictitious player answered correctly, in the other half, he answered incorrectly, by pointing a joystick either at the shell containing the ball or at the empty shell (factor Accuracy). In total, there were thus eight conditions: correct and erroneous observed responses in low difficulty trick trials, high difficulty trick trials, low difficulty no-trick trials and high difficulty no-trick trials. It was pseudo-randomized on which side (left or right) the ball was located at the start and the end of each trial and how long the trial lasted (two, three or four rotations).

Twelve trials of each Trial Type and Difficulty did not end with the player’s answer, but with the observer participants being asked which shell they thought the player would have chosen. After a static display of 400 ms of the final position of the shells, the respective question appeared (“Where will Daniel point the joystick?”) as well as the letters “L” and “R” for left and right under the corresponding shells. Trials ended after button press or after 2,700 ms if no response had been given until then. These prompts aimed to provide an insight into the observer’s expectations and were thus important to determine whether the intended manipulation of the observer participants’ expectations worked.

**Figure 1.**
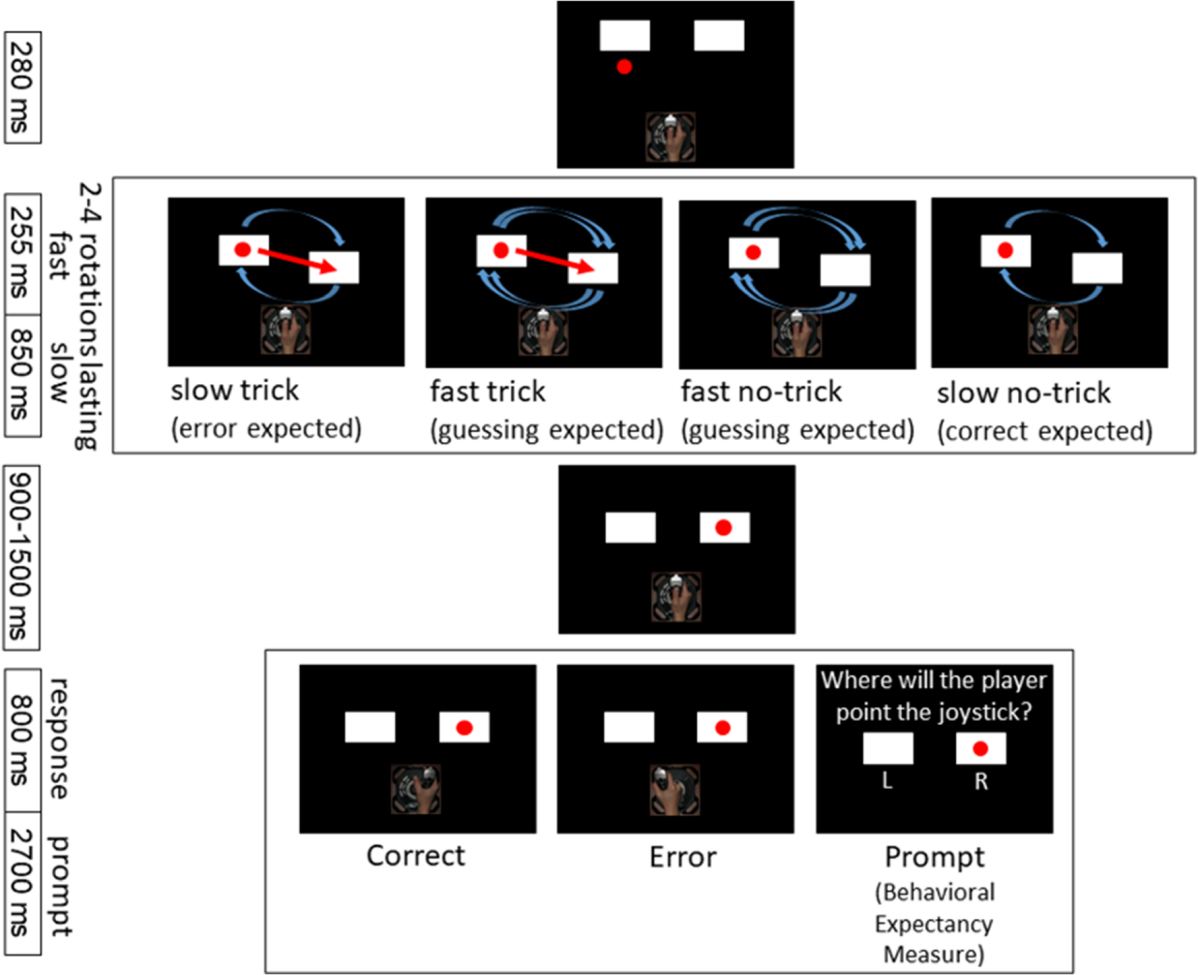
Time course of events in the experiment trials. There were eight conditions, low difficulty no-trick, low difficulty trick, high difficulty no-trick, and high difficulty trick, which either ended in a correct or an error response. Some trials ended not in a response by the observed player but in a prompt question to measure the observer’s expectancies.

A total of 420 trials were included in the EEG analysis, 105 trials for each combination of Trial Type and Difficulty. Forty-eight trials were included in the behavioral analysis: 12 of each Trial Type and Difficulty condition. The time course of the individual trials is shown in Figure 1.

### Empathy measure

Participants were asked to complete the German version of the Cambridge Behavior Scale (Baron-Cohen & Wheelwright, 2004; de Haen, n.d.), which is a measure of trait empathy. In a previous study (Bellebaum et al., 2020), we found that this empathy measure interacts with the experimental factors Trial Type and Accuracy of the paradigm that we (in an adapted version that additionally includes Difficulty) also applied in the present study, which is why we focused on this measure. The questionnaire contains 60 items, 20 of which are distractor items. Items consist of statements (e.g., “I really enjoy caring for other people”), which the participants can agree or disagree with using a four-point Likert scale ranging from “strongly agree” to “strongly disagree.” Items are scaled negatively or positively. Participants can score a maximum of two points per item. For positively scaled items, participants receive two points if they “strongly agree,” one point if they “slightly agree” and zero points if they “slightly disagree” or “strongly disagree.” For negatively scaled items, the scoring is reversed. Participants do not receive points for any answer on distractor items. Points are added and result in an empathy quotient (EQ) sum score that can range from 0 to 80.

### EEG Recording

Thirty passive scalp electrodes were applied according to the international 10-20 system (F7, F3, Fz, F4, F8, FT7, FC3, FCz, FC4, FT8, T7, C3, Cz, C4, T8, CP3, CPz, CP4, P7, P3, Pz, P4, P8, PO7, PO3, POz, PO4, PO8), and an electroencephalogram (EEG) was recorded throughout the experiment using a BrainAmp Standard amplifier (Brain Products, Munich, Germany) and the corresponding software (BrainVision Recorder, version 1.20.0506, Brain Products, Munich, Germany) at a sampling rate of 1,000 Hz. Electrodes were referenced to the average of two electrodes on the left and right mastoids. All impedances were kept below 5 kΩ.

### Procedure

Upon arrival in the laboratory, participants were informed about the experimental procedure and gave written informed consent to participate in the study. They were then asked to fill in a demographic questionnaire and the German version of the Cambridge Behavior Scale (Baron-Cohen & Wheelwright, 2004; de Haen, n.d.). After completion, we attached the EEG electrodes and participants were placed in front of a 1,920 * 1,080 px desktop monitor, and the experiment began. The experiment lasted about 45 minutes. The Stimulus presentation and response recording were controlled by Presentation Software (Version 20.0, Neurobehavioral Systems, Albany, CA).

### Data analyses

#### Behavioral data

We analyzed the responses to the prompt trials to determine how the observers’ expectancies concerning response accuracy of the player were modulated by the factors Trial Type and Difficulty. As in our previous studies (Kobza and Bellebaum, 2013; Bellebaum et al., 2020), we aimed to induce expectations of correct responses in no-trick trials and of error responses in trick trials, which were possibly less strong in high difficulty trials. We thus determined the proportion of the prompt trials in which the observer participants expected a correct response by the player for low difficulty and high difficulty no-trick and trick trials.

#### EEG data

EEG data were preprocessed using BrainVision Analyzer software, version 2.1 (Brain Products, Munich, Germany). Raw data were filtered with a 0.5-Hz high-pass and a 20-Hz low-pass filter. We then aimed to remove blink artefacts from the filtered raw signal. For this purpose, we performed an independent component analysis on single-subject EEG data. This analysis breaks down the raw data into temporally independent and spatially fixed components. We selected one component per participant that seemed to represent blink and vertical eye movement artifacts as observed in the vEOG electrode, as indicated by a symmetrical frontal distribution across the scalp. This component was then removed via independent component analysis back-transformation. For ERP analysis, we created segments of 800-ms length that started 200 ms before the observed choice (the time point when the joystick pointed to one of the shells). We performed a baseline correction using the average signal in the 200 ms before the observed choice. All segments in which a voltage step larger than 50 μV per ms occurred, in which highest and lowest data points differed by more than 100 μV or in which signals at any sample were higher than 100 μV or lower than −100 μV were excluded from further analysis automatically. On average, 3% of the error no-trick high difficulty segments (SD = 7%), 3% of the correct no-trick high difficulty segments (SD = 7%), 4% of the error no-trick low difficulty segments (SD = 8%), 4% of the correct no-trick low difficulty segments (SD = 7%), 2% of the error trick high difficulty segments (SD = 5%), 3% of the correct trick high difficulty segments (SD = 4%), 3% of the error trick low difficulty segments (SD = 4%), and 3% of the correct trick low difficulty segments (SD = 4%) were excluded. None of the participants lost more than 30% of all segments. Finally, single-subject averages were created for all eight conditions of the experiment. Data for each subject and condition were exported as text files and further processed in MATLAB, version R2017b (Mathworks, Natick, MA).

Based on the findings obtained in previous studies of our research group using this paradigm (Kobza & Bellebaum, 2013; Bellebaum et al., 2020), we expected that our experimental manipulation would affect a negative-going component in the ERPs between 250 and 420 ms after the observed choice. For this component, an interaction between the factors Trial Type and Accuracy (Kobza & Bellebaum, 2013; Bellebaum et al., 2020) as well as a modulation of this interaction by Empathy (Bellebaum et al., 2020) have been described. Thus, we investigated this component first. As in our previous studies, we calculated a peak-to-peak amplitude for the negative peak relative to a preceding positive peak. First, we pooled the signal over the electrodes Fz, FCz, and Cz, at which the component was particularly pronounced (see Bellebaum et al., 2020, for a similar procedure). We then calculated the most negative peak between 250 and 420 ms after the observed choice and subtracted the most positive peak in the preceding time window between 130 ms and the negative peak.

Contrary to our hypotheses, the ERPs seemed to be modulated by the experimental manipulations also in an earlier time window. Visual inspection of the signal at frontocentral electrodes suggested that the experimental factors modulated the first negative peak, that is, the N1 amplitude. Such an early modulation was not entirely unexpected: for the oERN, for example, as an ERP component reflecting the processing of observed actions, some variability has been found in studies concerning the latency with which it occurs. While it has mostly been reported to peak later than 200 ms after the response (van Schie et al., 2004; Miltner et al. 2004; Kobza & Bellebaum, 2013), there also are reports of shorter latencies (see Koban & Pourtois, 2014), with peaks as early as 150 ms after the onset of the observed response in some studies (Bates et al., 2005). It thus seems conceivable that a modulation of the processing of observed actions can take place in the N1 time window. To analyze this component, we also considered the pooled signal of three frontocentral electrodes (Fz, FCz, and Cz), because the component was also most pronounced frontocentrally (see topographic maps in the *Results* section). To score the component, we determined the most negative peak between 100 and 250 ms and subtracted the preceding most positive peak between 50 ms and the negative peak of the pooled signal. We will refer to this component as the early frontocentral negative component, while the component we analyzed first (see Bellebaum et al., 2020) will be referred to as the late frontocentral negative component.

#### Outlier detection

In each of the two EEG data sets (early component, late component) we determined participants whose peak-to-peak amplitude in at least one of the eight conditions differed by more than three standard deviations from the mean to identify outlier values in these dependent variables. The same criterion was used for the EQ sum score as continuous predictor variable. We found one participant with elevated scores for both dependent variables and excluded this participant from further data analysis.

#### Statistical analysis

The statistical analyses of the behavioral and EEG data were based on the following strategy. First, the behavioral data were analyzed to show if the task Difficulty factor, together with the Trial Type factor, affected observers’ expectancy in the intended way. Specifically, an interaction between the Trial Type and Difficulty factors was expected, with pronounced expectations concerning the accuracy of the observed action emerging only in low difficulty trials. In a second step, we analyzed to what extent Difficulty and Trial Type (due to their hypothesized effect on expectancy) affected the processing of observed actions, as reflected in the early and late frontocentral negative ERP components. If Trial Type and Difficulty interact in their effect on expectancy, the two factors also should interact in their effect on an ERP component reflecting expectancy. The focus in the analysis was thus on interactions involving these two factors. This analysis procedure established an indirect link between the behavioral (expectancy) data and the neurophysiological data. In addition, potential effects of the continuous factor Empathy were considered. We analyzed our data by means of linear mixed effects (LME) analyses using the lme4 statistical package (version 1.1-21) in R (version 3.5.3), because this type of analysis allows to include both categorical and continuous factors. All models were estimated using a restricted maximum likelihood approach, as proposed by Luke (2017). The R package lmerTest (version 3.1-0) was applied for evaluating significance in the models by using Satterthwaite approximation for the degrees of freedom. Effect sizes were calculated with the function anova_stats of the package sjstats (version 0.17.9).

For the behavioral data, we defined the dependent variable as the percentage of prompt trials in which participants indicated that they expected the player to choose the correct answer. We thus specified a model with percentage of expected correct answers as dependent variable and participants as a random-effect factor. Trial Type and Difficulty were defined as categorical fixed-effect predictors. We also included the random slopes of the categorical predictors by participants. As continuous factor we included Empathy (the EQ sum score). For the categorical factors, the levels of the Trial Type factor were recoded as +1 for trick and −1 for no-trick and for the factor Difficulty as +1 for low difficulty and −1 for high difficulty. The Empathy measures were mean-centered.

In the subsequent analysis of the EEG-data we analyzed the later as well as the earlier frontocentral negative ERP component. We thus specified two models, one for each of the components as dependent variable, that were similar to the model for the behavioral data, with Trial Type and Difficulty and the additional factor Accuracy as categorical fixed-effect predictors (modelling also their random slope by participants). Trial Type and Difficulty were recoded as in the model for the behavioral data (+1 = trick; −1 = no-trick; +1 = low difficulty; −1 = high difficulty). Accuracy was recoded as +1 for correct and −1 for error responses. The continuous factor, Empathy, and the model estimation were the same as in the model for the behavioral data. For all analyses the threshold for statistical significance was set to *p* < .05.

*Interactions*. Interactions were resolved in a step-wise manner according to Aiken, West, & Reno (1991): for every n-way interaction, we calculated slopes of the n-1-way interactions while one predictor was held constant. Significant interactions in these analyses were then resolved in the same way until all factors were resolved. For categorical factors, in accordance with the variable coding, we used 1 or −1 as constants. For the continuous factor Empathy, we shifted the variable by one standard deviation downward or upward from the mean (*M* – 1 *SD* or *M* + 1 *SD*) and calculated lower-level interactions while holding the continuous factor constant at low level empathy (low empathy, *M* – 1 *SD*) or high level empathy (high empathy, *M* + 1 *SD*).

*Analysis linking behavioral and ERP measures.* To explore whether there also was a direct relationship between expectancy and observed response processing, we planned to conduct follow-up analyses in case of a significant effect of the Trial Type and Difficulty factors on one of the ERP components. For this purpose, we calculated expectancy measures (concerning correct responses) for each of the conditions trick high difficulty, trick low difficulty, no-trick high difficulty, and no-trick low difficulty in every participant based on the prompt trials. These values indicated how strongly correct responses were expected. For error responses, the expectancy values were recalculated as 1 – expectancy of the correct response. The expectancy values were used as continuous factor Expectancy (mean-centered) in an LME model, including ERP component amplitudes as dependent variable. We included all participants that were included in the previous analyses in an additional outlier detection, based on the so-called Cook’s Distance. As Cook’s Distance measures the influence of single subjects on the model, this outlier detection method might be especially suitable for exploratory analyses where some aspects might not be perfectly controlled for (e.g., correlations between the predictors). Cook’s Distance analysis revealed one outlier participant that was excluded from the Expectancy analysis. To further test whether Empathy explained additional variance beyond the effect of expectancy, we calculated an exploratory Chi-Square test using the anova-function in R (from the package car, v 3.0-9) to compare the two models. This allowed us to determine whether a model including Expectancy and Empathy explained significantly more variance than a model including only Expectancy and thus, whether the frontocentral negative ERP component is further influenced by trait Empathy. For this comparison, both models were recalculated with a maximum likelihood approach.

## Results

Please find additional statistical data for the following LME analyses in the Supplementary Materials, including estimates, t-test statistics, standard errors, and confidence intervals for the data in the reported analyses.

### Behavioral analysis

**Figure 2.**
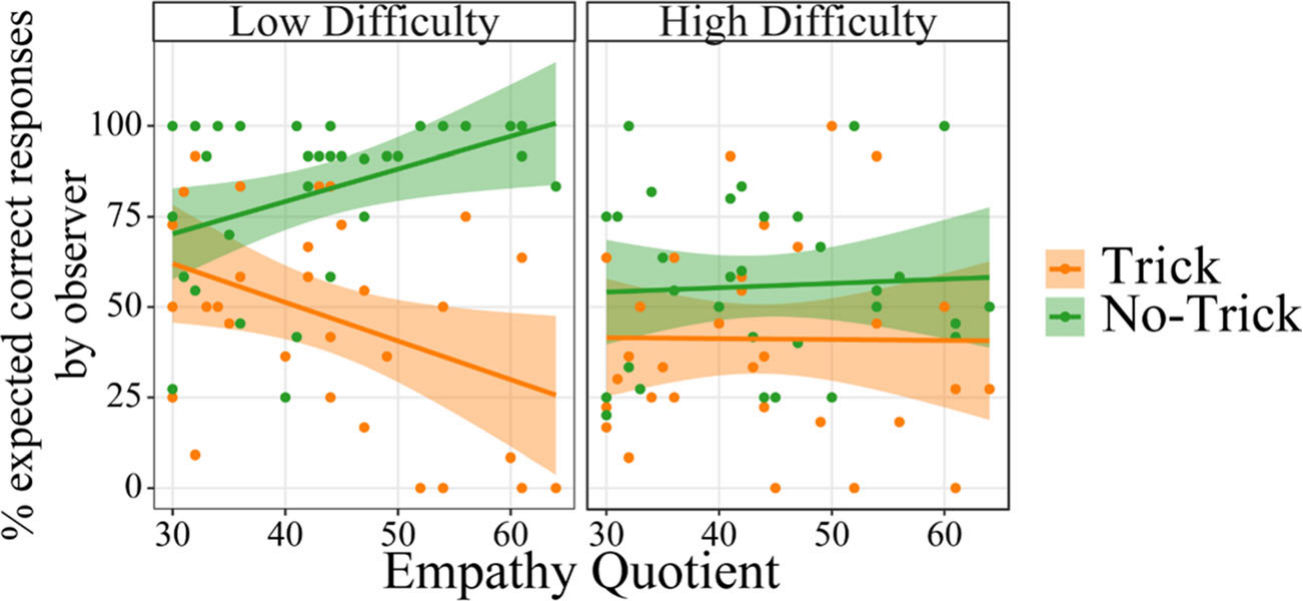
Behavioral data derived from prompt trials. Displayed is the percent of trials in which participants stated that they expected the player to answer correctly, modulated by trait Empathy, Trial Type, and trial Difficulty. Confidence intervals are displayed around the regression lines.

**Figure 3.**
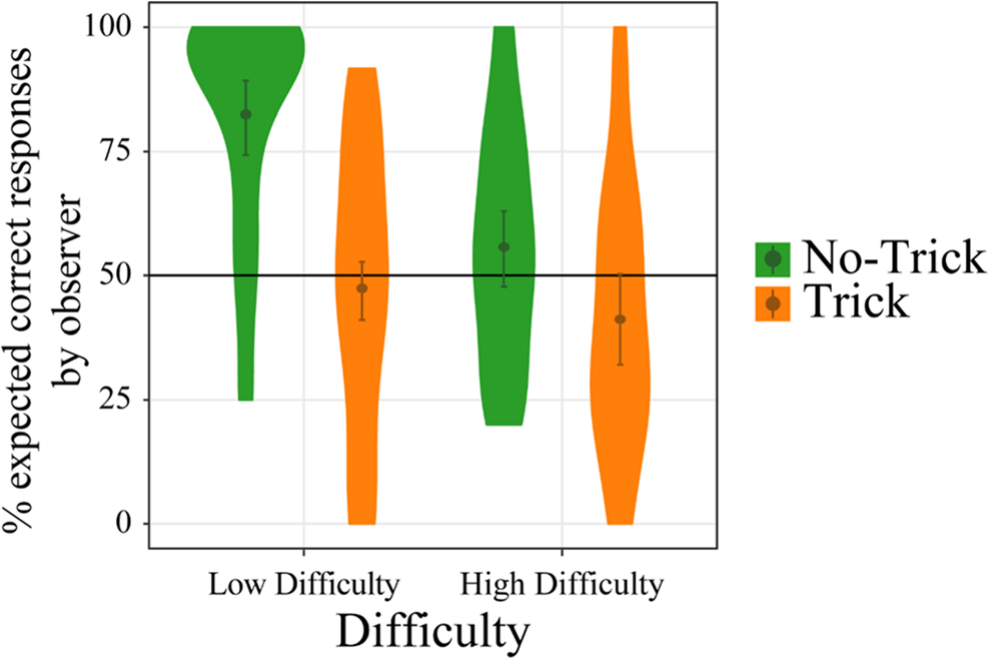
Interaction effect between Trial Type and trial Difficulty in behavioral data derived from prompt trials. The black line marks chance level (50%). Mean and confidence intervals are displayed within the respective violin plots.

The behavioral data reflecting the strength of the expectations of the observers are depicted in Figure 2. For the percentage of expected correct responses we found significant effects of Trial Type, *F*(1,31.00) = 25.94, *p* < 0.001, η_p_^2^ = 0.23, and Difficulty, *F*(1,31.00) = 11.55, *p* = 0.002, η_p_^2^ = 0.12. Trick trials resulted in lower expectation of correct answers (*b* = −12.41) than no-trick trials, as did high difficulty trials (*b* = 8.24) compared with low difficulty trials. Furthermore, a significant interaction between Trial Type and Difficulty emerged, *F*(1,30.99) = 8.85, *p* = 0.006, η_p_^2^ = 0.09. As can be seen in Figure 3, expectancy of correct responses was descriptively nearer to chance level in the high difficulty than low difficulty trials. A follow-up analysis to resolve the interaction of the two factors revealed, however, that the factor Difficulty was only significant for no-trick trials, F(1,55.91) = 20.18, *p* < 0.001, b = 13.35, not for trick trials (*p* = 0.299). In no-trick trials, correct answers were more expected in low difficulty trials than in high difficulty trials. Analyzing high and low difficulty trials separately, we found that Trial Type was significant for both low difficulty trials, *F*(1,55.75) = 35.63, *p* < 0.001, and high difficulty trials, *F*(1,55.75) = 5.98, *p* = 0.018, but the difference was less pronounced in high difficulty trials (*b* = −7.30 as opposed to *b* = −17.53). In both Difficulty conditions, correct answers were more expected in no-trick trials. As Difficulty interacted with Trial Type and the difference between trick and no-trick trials was less pronounced in high difficulty trials, we can conclude that the difficulty manipulation worked, as expected. Significant differences between low and high difficulty trials were found, however, only for no-trick, but not for trick trials.

We also found that Empathy interacted with Trial Type, *F*(1,31.00) = 4.59, *p* = 0.040, η_p_^2^ = 0.05, and with Trial Type and Difficulty, *F*(1,30.99) = 6.87, *p* = 0.013, η_p_^2^ = 0.07. We further investigated the interaction effects, including the continuous factor Empathy. We first considered the interaction between Empathy and Trial Type. Although the factor Trial Type modulated the answer for participants with both high and low empathy, *F*(1,31.00) = 26.14, *p* < 0.001, and *F*(1,31.00) = 4.32, *p* = 0.046, respectively, the effect was larger for high empathy participants (*b* = −17.66) than for low empathy participants (*b* = −7.17). For both groups, trick trials resulted in a lower expectation of correct answers than no-trick trials, but the effect was larger in participants with high empathy. Subsequently, we resolved the three-way interaction. A Trial Type by Empathy interaction was significant only for low difficulty trials, *F*(1,55.75) = 10.64, *p* = 0.002, not for high difficulty trials (*p* = 0.812). Further simple-slope analyses for low difficulty trials revealed that the factor Empathy was significant for low difficulty trick trials, *F*(1,40.83) = 5.31, *p* = 0.026, as well as low difficulty no-trick trials, *F*(1,45.66) = 5.34, *p* = 0.025. Higher empathy led to lower expectation of correct answers in low difficulty trick trials (*b* = −1.07) and to higher expectation of correct answers in low difficulty no-trick trials (*b* = 0.90). The main effect of Empathy and the remaining interaction did not reach significance (all *p* ≥ 0.786).

In summary, we found that the expectancy modulation by the factors Trial Type and Difficulty succeeded (Figure 3). Importantly, expectations were further modulated by empathy, with higher effects of the experimental factors on expectancy measures in high empathy participants. However, even for high empathy participants, we only found a significant effect of Difficulty in no-trick, but not in trick trials (Figure 2).

### EEG analysis

#### Late frontocentral negative component

**Figure 4.**
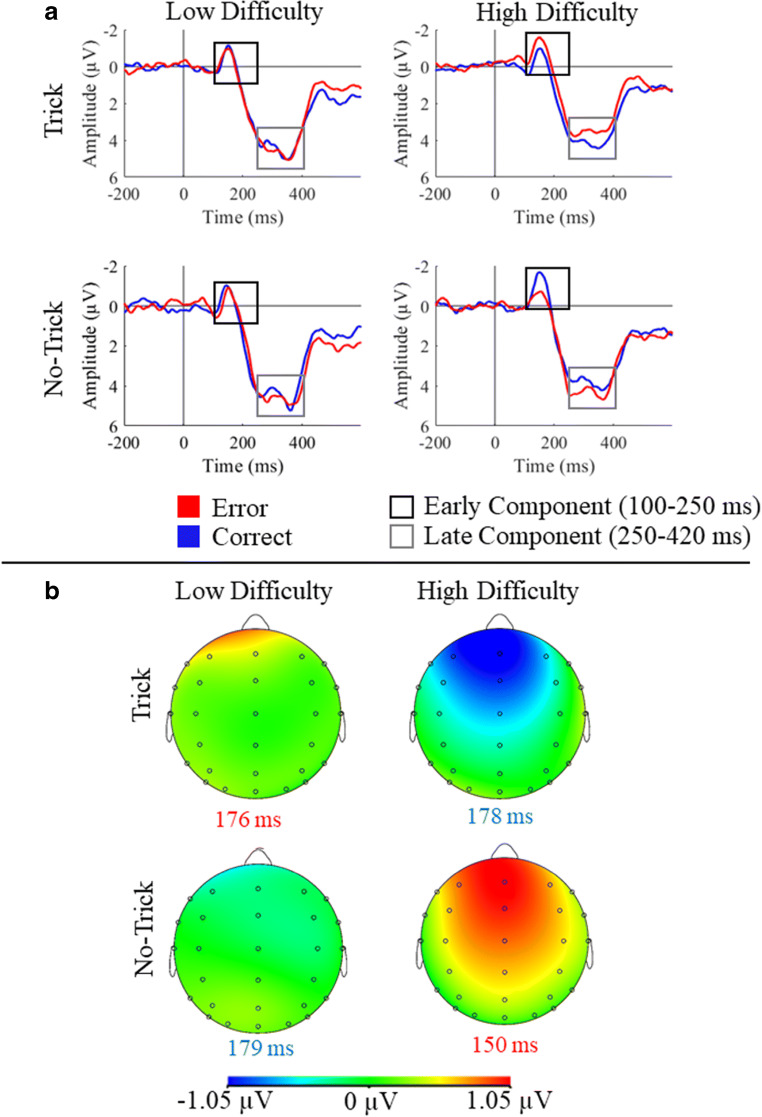
**A**. ERPs pooled over Fz, FCz, and Cz after observed correct and error responses for all combinations of the Trial Type and Difficulty conditions. The two analyzed components, early and late frontocentral negative component, are marked in the ERPs. **B**. Topography of the difference between the ERPs after error and correct responses at the maximum positive (low difficulty trick and high difficulty no trick) or the maximum negative (low difficulty no trick and high difficulty trick) peak of the difference between error and correct responses (between 150 and 180 ms) for the pooled signal of Fz, FCz, and Cz for both Trial Type and Difficulty conditions.

**Figure 5.**
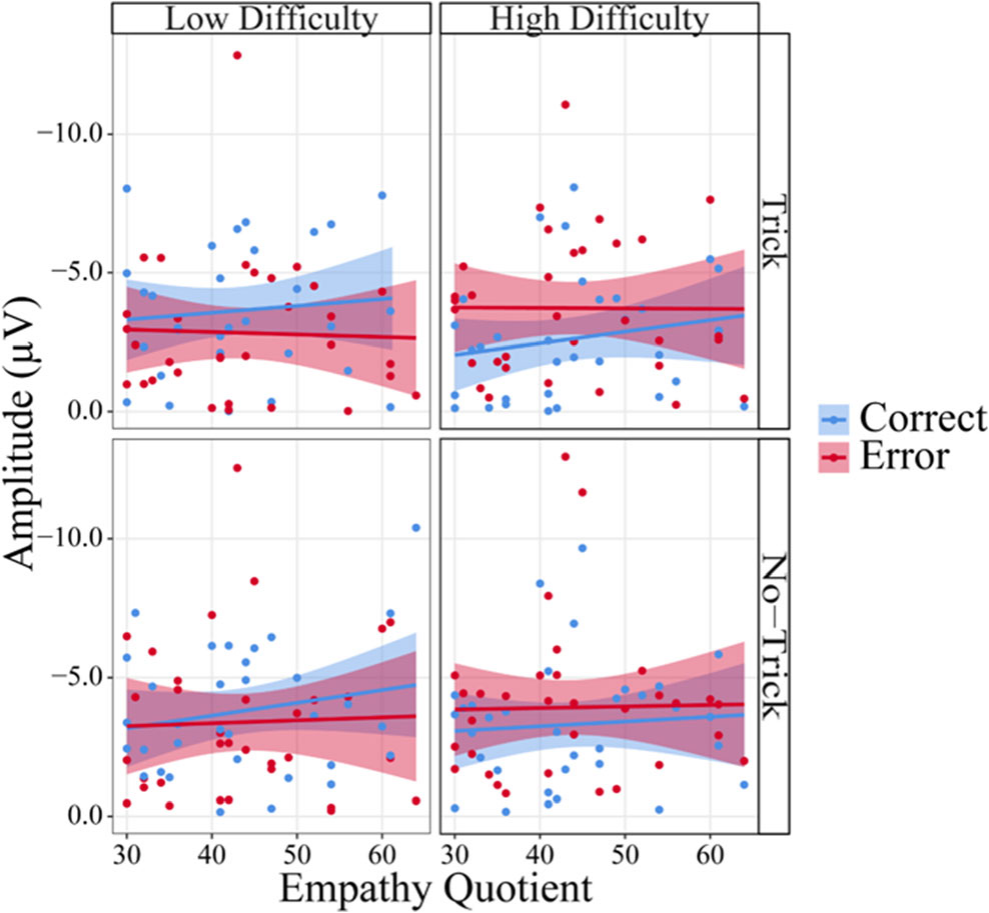
Peak-to-peak amplitudes of the late frontocentral negative component (250–420 ms) as a function of Trial Type, Difficulty, Accuracy and Empathy. Confidence intervals are displayed around the regression lines.

**Figure 6.**
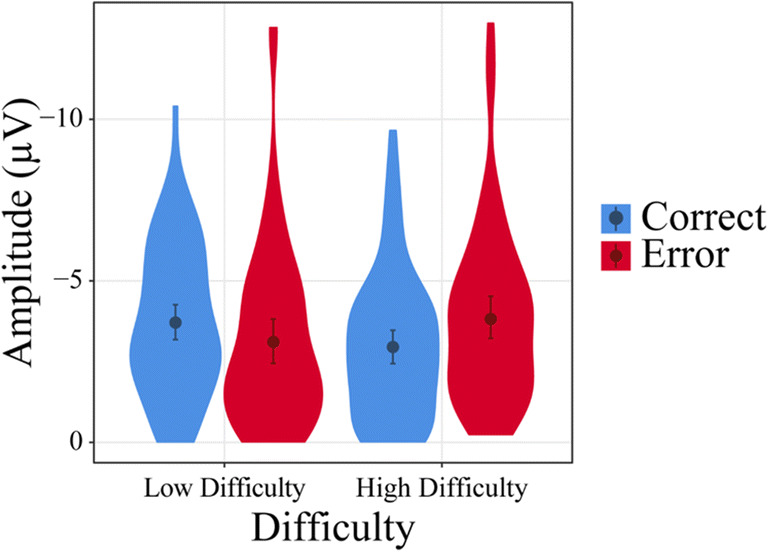
Interaction effect between Difficulty and Accuracy for the late frontocentral negative component (250–420 ms). Mean and confidence intervals are displayed within the respective violin plots.

The ERPs and their topography for the relationship between Trial Type, Difficulty and Accuracy are depicted in Figure 4. A display of the relationship between the four factors and the amplitude of the late frontocentral negative component, whose mean latency (across participants and conditions) was 335 ms (*SD* = 52 ms), is shown in Figure 5. The LME analysis did not reveal any main effects for the late negative component amplitude (all *p* ≥ 0.149). We found one significant interaction between Difficulty and Accuracy, *F*(1,124.84) = 11.77, *p* < 0.001 (for a visualization of this effect, see Figure 6). Follow-up analyses revealed that Accuracy modulated the late negative component only in high difficulty trials, *F*(1,59.99) = 4.75, *p* = 0.033, η_p_^2^ = 0.06, not in low difficulty trials (*p* = 0.130). For high difficulty trials, errors elicited a larger amplitude (*b* = 0.42) than correct responses. We did not find any other interaction effects (all *p* ≥ 0.323).

In summary, we did not find the expected modulation of ERPs in the late frontocentral negative ERP component (Figure 5), as the result pattern did not mirror the expectancy modulation by the factors Trial Type and Difficulty in the form of an interaction between the factors. Instead we found a selective modulation of the late component by Accuracy in high difficulty trials (Figure 6).

#### Early frontocentral negative component

**Figure 7.**
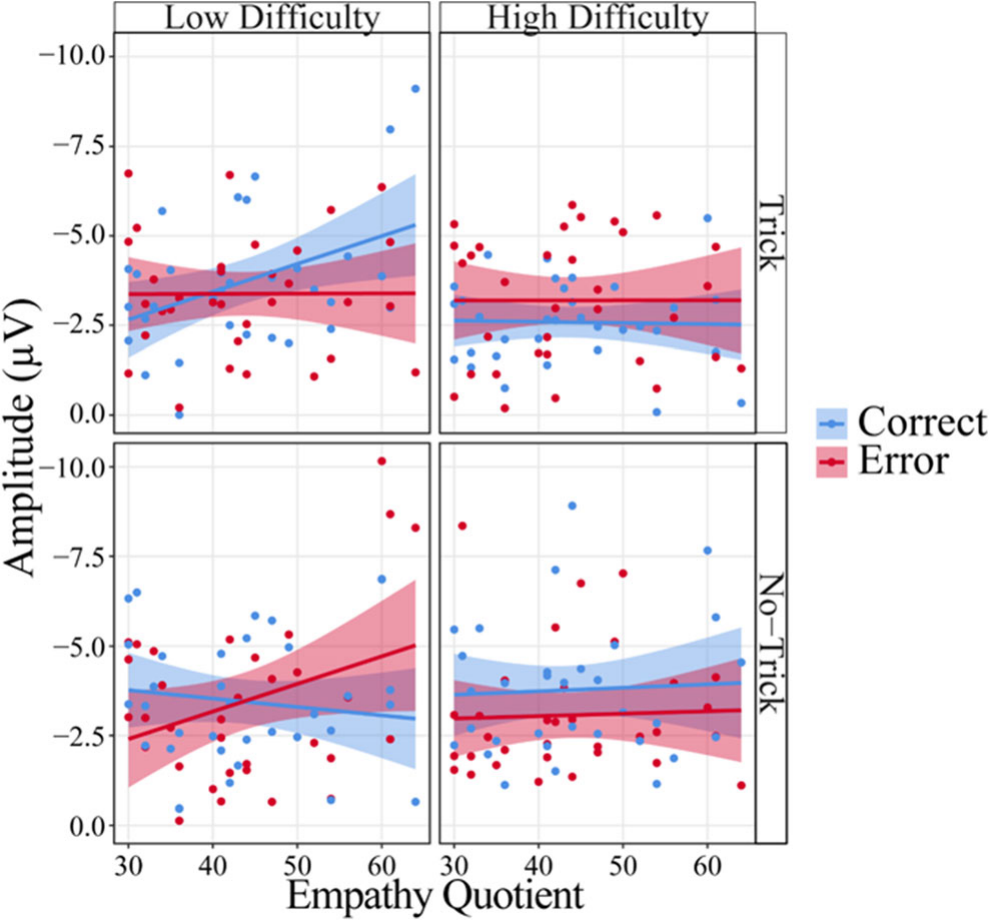
Peak-to-peak amplitudes of the early frontocentral negative component as a function of Trial Type, Difficulty, Accuracy, and Empathy. Confidence intervals are displayed around the regression lines.

Figure 7 shows the relation between the four factors and the amplitude of the early frontocentral negative component. The mean latency of this component (across participants and conditions) was 159 ms (*SD* = 27 ms). The LME analysis on this component’s amplitude revealed no significant main effects and no two-way interactions (all *p* ≥ 0.069). Instead we found two three-way interactions, one between Empathy, Trial Type and Accuracy, *F*(1, 186.02) = 6.04, *p* = 0.015, η_p_^2^ = 0.03, and one between Trial Type, Difficulty and Accuracy, *F*(1,186.02) = 5.64, *p* = 0.019, η_p_^2^ = 0.03, but no other three-way-interactions (all *p* ≥ 0.524). Because we also found a significant four-way interaction between all four factors—Empathy, Trial Type, Difficulty, and Accuracy, *F*(1, 186.02) = 6.96, *p* = 0.009, η_p_^2^ = 0.03, we focused on the resolution of this highest-order interaction. We thus conducted two further LME-analyses separately: one for low difficulty and one for high difficulty trials. A significant three-way interaction between Trial Type, Accuracy, and Empathy emerged for low difficulty trials, *F*(1,186.01) = 12.98, *p* < 0.001, but not for high difficulty trials (*p* = 0.898). In the resolution of the interaction for low difficulty trials, an Accuracy by Empathy interaction was found for both low difficulty trick, *F*(1,194.18) = 4.83, *p* = 0.029, and low difficulty no-trick trials, *F*(1,194.08) = 8.20, *p* = 0.005. For low difficulty trick trials, a significant effect of empathy was found only for correct, *F*(1,77.83) = 7.02, *p* = 0.010, but not for error responses (*p* = 0.984); for no-trick trials the pattern was reversed: an effect of Empathy was found for error, *F*(1,52.71) = 4.56, *p* = 0.037, but not for correct trials (*p* = 0.492). Higher empathy resulted in more negative amplitudes for low difficulty correct trick trials (*b* = −0.08) and for low difficulty error no-trick trials (*b* = −0.08). Analyzing the high difficulty trials separately, no three-way interaction emerged (*p* = 0.898), but an interaction between Trial Type and Accuracy could be found, *F*(1, 186.01) = 7.23, *p* = 0.008. A significant effect of Accuracy emerged only for high difficulty no-trick trials, *F*(1,194.20) = 4.10, *p* = 0.044, where correct responses led to higher amplitudes (*b* = −0.35), although we found a trend for an Accuracy effect also in high difficulty trick trials, *F*(1,194.16) = 3.07, *p* = 0.081, where errors led to higher amplitudes (*b* = 0.31).

In summary, we found a modulation in an earlier time window (around the N1) similar to the one we expected. Consistent with the behavioral results for expectancy formation, we found that ERPs were modulated by an interaction of Empathy, Trial Type, and Accuracy for low difficulty trials only, where Empathy seemed to be important particularly for the processing of those events that were considered unexpected (see the low difficulty grids in Figure 7).

#### Effects of expectancy and empathy on the early frontocentral negative component

**Figure 8.**
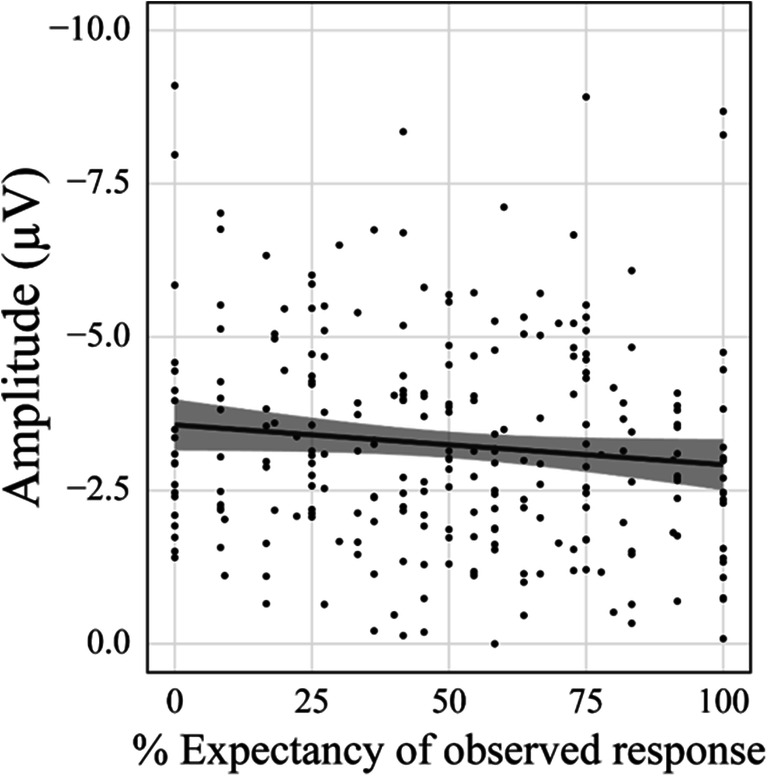
Effect of Expectancy on the early frontocentral negative component. Confidence intervals are displayed around the regression lines.

After we found that Trial Type and Difficulty affected the early frontocentral negative ERP component, we aimed to explore the relationship between expectancy and the amplitude directly (see *Methods* section). We found a main effect of Expectancy, *F*(1,220.43) = 4.57, *p* = 0.034, η_p_^2^ = 0.02, *b* = 0.65. For higher Expectancy values, amplitudes were smaller (Figure 8). We further conducted an analysis in which we compared a model including only Expectancy to a model including Expectancy and Empathy. This analysis must be considered exploratory, because empathy predicted expectancy in our experiment, as was revealed by the behavioral data analysis. The two factors in the Expectancy x Empathy model were thus not independent. A model including both predictors did not explain significantly more variance than a model including only Expectancy, Χ^2^(2) = 0.07, *p* = 0.967. In summary, the measured Expectancy values functioned as predictors for the amplitude of the early frontocentral negative component. Empathy did not account for significantly more variance if Expectancy was already included as a predictor.

## Discussion

In this study, we investigated the role of expectations in the processing of observed actions and a potentially mediating effect of empathy. To this end, we had participants observe a person perform correct or error responses in a two-shell-game. Expectancy was modulated by two factors that allowed to distinguish between effects of vicarious errors and expectancy. We found that our manipulation of the expectancy of the observed response succeeded. We also found an effect of empathy on the strength of the expectations. Concerning the neurophysiological processing of observed responses, there was evidence that the amplitude of a frontocentral negative ERP component time-locked to observed responses was mainly driven by the expectancy of the responses. Surprisingly, this pattern was found in the N1 time window and thus earlier than the ERP components that have been linked to observed response processing in previous studies (Kobza and Bellebaum, 2013; Bellebaum et al., 2020; van Schie et al., 2004; see also Koban & Pourtois, 2014).

### Behavioral measures of expectancy

We measured self-reported expectancies concerning the observed response separately for each condition. This assessment served to verify whether our experimental manipulations affected participants’ expectancies in the intended way, which was an important prerequisite for the interpretation of the ERP data. We found a modulation by a false-belief condition (factor Trial Type), in accordance with previous studies applying the same paradigm (Bellebaum et al., 2020; Kobza and Bellebaum, 2013). Moreover, the newly introduced factor task Difficulty affected participants’ expectations in that the difference between conditions with true and false belief was less pronounced for trials with high difficulty. Analyzing true- and false-belief conditions separately, we found a modulation by task Difficulty only in the true-belief, but not in the false-belief condition. One reason seems to be that expectations in low difficulty trials with a false-belief were less strong than expectations for low difficulty true-belief trials. Expectations for false-belief conditions seem to be harder to form (the same effect was found in previous studies employing this paradigm, see Bellebaum et al., 2020; Kobza & Bellebaum, 2013; as well as in studies using false-belief tasks, see Birch & Bloom, 2007; Wellman, Cross, & Watson, 2001), and with the additional factor Difficulty, this might have led to smaller differences between the Difficulty conditions. We also found that Empathy influenced expectancy formation in low difficulty conditions: expectancies were formed most consistently in high empathy participants. Bellebaum et al. (2020) did not find a modulation of expectancy by empathy using the same paradigm. In this previous study there was little interindividual variance in the expectancies, which clearly differed between false- and true-belief conditions. The introduction of the task difficulty variation in the present study may have led to more variance, so that empathy may have plaid a more important role for expectancy generation.

It is important to keep in mind that expectancy was assessed based on 12 prompt trials per condition only, so the derived expectancy values do not reflect expectations on a single trial basis. Also, it has to be noted that the prompt trials primarily served to check if the expectancy manipulation succeeded. While the LME analysis suggests an interesting modulation of expectancy by empathy, the behavioral results should be interpreted with caution.

### Latency of expectancy and empathy effects on the processing of observed actions

An important difference between the present study and our previous studies with the same paradigm (Kobza and Bellebaum, 2013; Bellebaum et al., 2020) is that the modulation of the ERPs by Expectancy and Empathy occurred much earlier after the observed response in the present study. The component also had a lower latency than the oERN, at least for most of the studies investigating this component (Carp et al., 2009; de Bruijn & von Rhein, 2012; Miltner et al., 2004; van Schie et al., 2004); it occurred between 100 and 250 ms (mean 159 ms) and thus in the latency range of the N100. Nevertheless, we have reason to believe that this early modulation reflects an ERP component resembling other components that have been linked to the processing of observed responses. Firstly, the topography of the relative negativity in the ERPs for unexpected events showed a fronto-central maximum (Figure 4) and is thus not only consistent with the typical topography of the oERN, but also with that of the ERN and the feedback-related negativity, which are all related to performance monitoring (Falkenstein et al., 1991; Gehring et al., 2012; Gehring & Willoughby, 2002; Miltner et al., 2004; van Schie et al., 2004). Second, the modulation by expectancy and/or empathy is comparable to modulations of monitoring-related ERPs found in previous studies (Bellebaum et al., 2020; Ferdinand et al., 2012; Kobza & Bellebaum, 2013; Wessel et al., 2012). Particularly, these results correspond to those of Kobza and Bellebaum (2013) and Bellebaum et al. (2020), who applied the same paradigm but found the effect in a later time window. Third, and most important, the latency of the oERN and related components appears to differ depending on the task. For a Go/NoGo-Task an oERN was observed as early as 150 ms after the observation of errors in NoGo trials which corresponds to the latency range of the present study (Bates et al., 2005; Koban et al., 2010). This earlier latency has been linked to the lower complexity compared with a Flanker task (Koban & Pourtois, 2014).

The question remains, however, why the modulation in the present study occurred so early. The main difference between the present and our previous studies (Bellebaum et al., 2020; Kobza & Bellebaum, 2013) is that we used a block design for trick and no-trick trials, so that participants knew beforehand whether the observed person had a true or a false belief. In blocks with trick trials, for example, they knew that the observed person was more likely to point to the empty shell, performing an error. If trick and no-trick trials alternate trial-by-trial, as in our previous studies, expectation formation probably takes more time. We believe that this early expectation formation enabled faster processing and thus earlier ERP modulations. We therefore discuss the early ERP modulation in the following sections and relate it to findings from the literature, where mostly later components were analyzed, but emphasize that these results should be interpreted with caution as our hypothesis was related to a modulation in a later time window.

### Effects of expectation on observed error processing

Consistent with the previous results we obtained with this paradigm (Kobza and Bellebaum, 2013; Bellebaum et al., 2020), we found that the amplitude of a negative ERP component following an observed response was modulated by expectancy, although this modulation occurred earlier than in the previous studies In participants who developed strong expectations, the least expected events, that is, correct responses in low difficulty trials with a false-belief and error responses in low difficulty trials with a true belief, elicited the highest amplitudes.

Importantly, we found a modulation not only by the false-belief condition, but also by the new factor task Difficulty. If the ERP component had only reflected the false-belief condition and not task difficulty, the modulation could have been ascribed to vicarious error processing, as errors from the perspective of the observed person were the same in both Difficulty conditions. As this is not the case, we conclude that the ERP modulation codes expectancy rather than vicarious errors, which corresponds with the interpretation of Kobza and Bellebaum (2013) and Bellebaum et al. (2020).

This supports theories on the role of expectancy for amplitudes of ERP components generated by the ACC or pMFC, stating that these components primarily code unexpected events irrespective of valence (Alexander & Brown, 2011). It also matches other recent results. Wessel et al. (2012) found a common neural generator, namely the pMFC, of both the ERN and the novelty-related frontocentral N2, suggesting an overlap of the neural correlates of error and surprise processing. Ferdinand et al. (2012) showed that the FRN was elicited not only by unexpected negative, but also by unexpected positive feedback. For observed actions, Schiffer, Krause, & Schubotz (2014) reported activity in the medial prefrontal cortex after unexpected incorrect as well as unexpected correct responses in a functional magnetic resonance imaging study (see also Wang et al., 2015). Our study thus adds to existing evidence that activity in the medial prefrontal cortex and ACC in response to different events is critically modulated by the expectancy of these events.

It has to be noted that, in contrast to our hypotheses, the ERP amplitude pattern was reversed in high difficulty trials compared with low difficulty trials. Amplitudes were enhanced for correct actions in true-belief and erroneous actions in false-belief trials, respectively, which is not in accordance with the behavioral expectancy measures. We suspect that although participants formed explicit expectations, at least in the true-belief condition, further implicit expectations might have played a role, too. Multiple studies suggest that responses to errors or infrequent events lead to increased attention to the source of the (prediction) error (Notebaert, Houtman, Opstal, Gevers, Fias, & Verguts, 2009; Steinhauser & Andersen, 2019; Wessel, 2018). In our study, trick and no-trick trials were presented in separate blocks, while low difficulty and high difficulty trials were mixed within one block. This means that for every trial, the observer participants only had to consider whether the trial was difficult (high difficulty) or not (low difficulty) for their expectancy of the correct response, so that high difficulty and low difficulty trials were directly compared to each other. As participants had to focus on differentiating high and low difficulty trials, their attention might have been relocated to this comparison as opposed to absolute probabilities. The modulations observed might thus code for the comparison between high and low difficult trials, meaning that the likelihood of the actually more expected responses might be implicitly underestimated in high difficulty trials, resulting in the observed reverse pattern.

The exact mechanisms of expectancy formation, especially concerning explicit and implicit expectancies that might have played a role in our study, are not completely understood. In a study in which participants observed erroneous everyday actions in a virtual reality setting, Pezzetta, Nicolardi, Tidoni, & Aglioti (2018) found a modulation of the ERPs by the accuracy of the observed response, with higher oERN amplitudes for errors, also when errors occurred more frequently than correct actions. A reason for this could be that the authors used simple everyday actions which might generally be expected to be performed correctly. Furthermore, other studies suggest that the way events are processed is not entirely determined by their frequency. Several studies found differences between the processing of negative and positive feedback processing even if these events were equally probable (Wang et al., 2015; Yeung et al., 2005). These findings have been ascribed to an overoptimistic bias of participants to expect correct responses or positive outcomes more strongly, especially for own behavior (Oliveira, McDonald, & Goodman, 2007). Ferdinand et al. (2012) found comparable amplitudes for unexpected positive and negative feedback in the FRN, but observed an effect of valence in the P300, with positive feedback eliciting larger amplitudes than negative feedback. This difference in early and later processing resembles the difference between the early and the late frontocentral negative component in high difficulty trials in this study, with the early component being modulated by expectancy and the late component being modulated by valence. Also, Ferdinand et al. describe a difference between actual expectations (more than half of the participants believed that negative feedback was more frequent when asked after the experiment, only less than a quarter thought that positive feedback was more frequent) and FRN amplitudes, again suggesting that other, less conscious processes play a role in early processing when outcomes are uncertain. Moreover, other factors apart from expectancy may also affect neural indices of performance monitoring: Maier and Steinhauser (2016) found that active responders’ ERN was modulated by error significance rather than error probability.

### Effects of empathy on observed error processing

Another similarity between the present study and our previous work on action observation is that empathy affected the processing of observed responses (Bellebaum et al., 2020). In the present study, this effect was restricted to the low difficulty condition, where only for highly empathic participants ERP amplitudes were higher for unexpected than expected events. Figure 7 shows that the processing of unexpected events (correct responses in low difficulty trick trials and error responses in low difficulty no-trick trials) was exclusively modulated by empathy. As outlined in the introduction, our previous finding might have reflected vicarious error processing, as in the false-belief condition of our task correct responses were errors from the observed person’s view. However, by showing that the empathy effect is restricted to low difficulty trials, this interpretation appears to become less likely, as correct responses in high difficulty trick-trials are also errors for the observed person. Our behavioral finding that only the expectancies in low difficulty trials were modulated by empathy instead appears to suggest that expectancy plays a modulatory role for the influence of empathy on the processing of observed actions.

### Empathy, expectation, and the processing of observed actions

Our results show an effect of empathy on both the expectancy data and the ERPs. In an additional exploratory analysis, we examined the relationship between expectation, empathy and the ERP amplitudes in the eight conditions in one LME analysis. We found a positive relationship between expectancy and ERP amplitude, which was not modulated by empathy. We also found that including empathy as a predictor did not explain significantly more variance than using expectancy alone. Keeping in mind that empathy and expectancy were correlated (see above), these results suggest that empathy did not influence ERPs in the present study beyond the effect it had on expectancy formation. This might be an indication that empathy did not directly influence ERPs but via a positive effect on the expectancy formation. Several studies addressed the relationship between trait empathy and the processing of observed actions or action outcomes (Bellebaum et al., 2020; Fukushima & Hiraki, 2006, 2009; Kobza, Thoma, Daum, & Bellebaum, 2011; Newman-Norlund et al., 2009; Shane et al., 2009), but expectancy was rarely taken into consideration, which might be one reason for the inconsistency in the findings. In the context of outcome processing, Lockwood et al. (2015) described that a subregion of the ACC specifically predicted other’s rewards in highly empathic participants, whereas activity in that region was comparable for other’s and own rewards in low empathizers. These findings may mean that empathy facilitates the generation of predictions based on other’s assumed mental states by helping to see an event from the perspective of the observed person. At the same time, positive linear relationships between empathy and expectancy are not always found. In our previous related study (Bellebaum et al., 2020), the observers could more easily predict what action the observed person was about to perform as task difficulty was not varied. Accordingly, participants developed strong expectations regarding the observed person’s response with little interindividual variability and thus little room for a modulation by empathy. Brown and Brüne (2012) suggest that predictions in social contexts may depend on similar processes as predictions in nonsocial contexts, but that additional (social) factors play a role only in social contexts. Extending this assumption based on the present findings, it might be that the more the context is dominated by social factors, the more predictions might be modulated by trait empathy. This idea finds some support by findings of Fukushima and Hiraki (2009), who reported that empathy affected the observer FRN only if participants observed humans, not if they observed PC programs.

### Limitations

Due to a relatively large number of exclusions, we analyzed a smaller sample than planned originally. Investigating individual differences in a small sample can lead to false-positive results. LME analysis allows for the inclusion of random effects, so that further interindividual differences besides empathy are at least partly subtracted from the results (i.e., noise is removed; Baayen, Davidson, & Bates, 2008). Nevertheless, it cannot be excluded that the results in the present study represent a false positive result and thus they should be interpreted with caution. Future studies should aim for an increased sample size when investigating effects of empathy on error processing.

## Conclusions

Applying a complex action observation task with true- and false-belief conditions, we found that expectancy, not vicarious errors, was reflected in ERPs time-locked to the observed response, although in an earlier time window as previously suspected. Both the expectancy of the observed action and the ERPs following the observed action were modulated by empathy. We suggest that trait empathy facilitates the processing of stimuli and events from another person’s perspective by facilitating expectancy formation. Furthermore, empathy seems to be necessary for expectancy formation only for specific contexts in which social factors dominate. The results found in this study, specifically regarding the indirect influence of empathy on performance monitoring via expectation generation, could help to understand the nature of the problems in social interactions typically found in patients with reduced empathic abilities and may have implications for therapeutic approaches. For example, adding information that makes it easier for these persons to predict and understand others’ actions may improve their social skills. Further research needs to investigate the factors that determine the timing of expectancy and empathy modulations in the processing of observed actions.

## Supplementary Information

ESM 1 (DOCX 43 kb)
