# Supplementary material for: Effects of trait empathy and expectation on the processing of observed actions
Source: Cogn Affect Behav Neurosci. 2020 Dec 9;21(1):156–71. doi: 10.3758/s13415-020-00857-7 (PMC7994233; doi:10.3758/s13415-020-00857-7)
Supplement: Supplementary file 1 — (DOCX 43 kb) [file 13415_2020_857_MOESM1_ESM.docx]

Supplementary Materials

Additional statistical data for all three LME analysis (behavioral, late frontocentral negative component, and early frontocentral negative component).

**Table A1**

*Statistical data for behavioral LME analysis*

| Effect | Estimate (*b*) | Std. error | *df* | *t*-value | *p*-value | CI 2.5% | CI 97.5% |
| --- | --- | --- | --- | --- | --- | --- | --- |
| (Intercept) | 56.70 | 1.97 | 30.99 | 28.85 | **<.001** | 52.68 | 60.79 |
| Trial Type | -12.41 | 2.44 | 31.00 | -5.09 | **<.001** | -16.87 | -7.44 |
| Difficulty | 8.24 | 2.42 | 31.00 | 3.40 | **.002** | 3.43 | 12.94 |
| Empathy | -0.02 | 0.20 | 30.99 | -0.10 | .922 | -0.37 | 0.37 |
| Trial Type x Difficulty | -5.12 | 1.72 | 30.99 | -2.97 | **.006** | -8.58 | -1.72 |
| Trial Type x Empathy | -0.53 | 0.25 | 31.00 | -2.14 | **.040** | -1.06 | -0.05 |
| Difficulty x Empathy | -0.07 | 0.24 | 31.00 | -0.27 | .786 | -0.57 | 0.44 |
| Trial Type x Difficulty x Empathy | -0.46 | 0.17 | 30.99 | -2.62 | **.014** | -0.78 | -0.07 |

*Note*. Degrees of Freedom (*df*), *t*- and *p*-values as well as estimates (*b*) based on a restricted maximum likelihood approach, as proposed by Luke (2017) for the Trial Type x Difficulty x Empathy LME analysis on the behavioral expectancy data. Satterthwaite approximation was used for the degrees of freedom. Significant values are displayed in bold font.

**Table A2**

*Statistical data for late frontocentral negative component LME analysis*

| Effect | Estimate (*b)* | Std. error | *df* | *t*-value | *p*-value | CI 2.5% | CI 97.5% |
| --- | --- | --- | --- | --- | --- | --- | --- |
| (Intercept) | -3.40 | 0.31 | 17.13 | -11.02 | **< .001** | -4.02 | -2.83 |
| Trial Type | 0.21 | 0.14 | 30.63 | 1.48 | .149 | -0.10 | 0.50 |
| Difficulty | -0.01 | 0.11 | 30.13 | -0.13 | .900 | -0.23 | 0.21 |
| Accuracy | 0.06 | 0.16 | 31.28 | 0.39 | .702 | -0.26 | 0.40 |
| Empathy | -0.03 | 0.03 | 17.67 | -0.96 | .350 | -0.10 | 0.04 |
| Trial Type x Difficulty | -0.03 | 0.10 | 124.83 | -0.24 | .810 | -0.20 | 0.17 |
| Trial Type x Accuracy | 0.01 | 0.10 | 124.83 | 0.08 | .934 | -0.18 | 0.23 |
| Trial Type x Empathy | 0.00 | 0.01 | 31.23 | 0.23 | .820 | -0.02 | 0.03 |
| Difficulty x Accuracy | -0.36 | 0.10 | 124.84 | -3.43 | **< .001** | -0.58 | -0.17 |
| Difficulty x Empathy | 0.00 | 0.01 | 30.34 | -0.02 | .985 | -0.02 | 0.02 |
| Accuracy x Empathy | -0.01 | 0.02 | 29.26 | -0.85 | .400 | -0.04 | 0.02 |
| Trial Type x Difficulty x Accuracy | -0.10 | 0.10 | 124.84 | -0.99 | .323 | -0.30 | 0.09 |
| Trial Type x Difficulty x Empathy | 0.01 | 0.01 | 126.20 | 0.77 | .442 | -0.01 | 0.03 |
| Trial Type x Accuracy x Empathy | 0.00 | 0.01 | 126.20 | -0.26 | .796 | -0.02 | 0.02 |
| Difficulty x Accuracy x Empathy | 0.00 | 0.01 | 126.20 | -0.10 | .919 | -0.02 | 0.02 |
| Trial Type x Difficulty x Accuracy x Empathy | 0.00 | 0.01 | 126.20 | 0.46 | .644 | -0.02 | 0.03 |

*Note*. Degrees of Freedom (*df*), *t*- and *p*-values as well as estimates (*b*) based on a restricted maximum likelihood approach, as proposed by Luke (2017) for the Trial Type x Difficulty x Accuracy x Empathy LME analysis on the late frontocentral negative component. Satterthwaite approximation was used for the degrees of freedom. Significant values are displayed in bold font.

**Table A3**

*Statistical data for early frontocentral negative component LME analysis*

| Effect | Estimate (*b)* | Std. error | *df* | *t*-value | *p*-value | CI 2.5% | CI 97.5% |
| --- | --- | --- | --- | --- | --- | --- | --- |
| (Intercept) | -3.33 | 0.20 | 31.03 | -16.23 | **< .001** | -3.75 | -2.95 |
| Trial Type | 0.11 | 0.09 | 68.56 | 1.16 | .251 | -0.06 | 0.30 |
| Difficulty | -0.17 | 0.09 | 45.05 | -1.86 | .069 | -0.35 | 0.03 |
| Accuracy | -0.05 | 0.09 | 89.50 | -0.61 | .543 | -0.23 | 0.13 |
| Empathy | -0.02 | 0.02 | 31.03 | -0.88 | .385 | -0.06 | 0.02 |
| Trial Type x Difficulty | -0.16 | 0.09 | 186.02 | -1.81 | .072 | -0.31 | 0.01 |
| Trial Type x Accuracy | 0.12 | 0.09 | 186.02 | 1.43 | .155 | -0.06 | 0.29 |
| Trial Type x Empathy | 0.00 | 0.01 | 68.56 | -0.07 | .945 | -0.02 | 0.02 |
| Difficulty x Accuracy | -0.03 | 0.09 | 186.02 | -0.35 | .726 | -0.20 | 0.14 |
| Difficulty x Empathy | -0.01 | 0.01 | 45.05 | -1.60 | .116 | -0.03 | 0.00 |
| Accuracy x Empathy | 0.00 | 0.01 | 89.50 | 0.33 | .741 | -0.02 | 0.02 |
| Trial Type x Difficulty x Accuracy | -0.21 | 0.09 | 186.02 | -2.37 | **.019** | -0.38 | -0.04 |
| Trial Type x Difficulty x Empathy | -0.01 | 0.01 | 186.02 | -0.64 | .524 | -0.02 | 0.01 |
| Trial Type x Accuracy x Empathy | -0.02 | 0.01 | 186.02 | -2.46 | **.015** | -0.04 | 0.00 |
| Difficulty x Accuracy x Empathy | 0.00 | 0.01 | 186.02 | 0.33 | .743 | -0.01 | 0.02 |
| Trial Type x Difficulty x Accuracy x Empathy | -0.02 | 0.01 | 186.02 | -2.64 | **.009** | -0.04 | 0.00 |

*Note*. Degrees of Freedom (*df*), *t*- and *p*-values as well as estimates (*b*) based on a restricted maximum likelihood approach, as proposed by Luke (2017) for the Trial Type x Difficulty x Accuracy x Empathy LME analysis on the early frontocentral negative component. Satterthwaite approximation was used for the degrees of freedom. Significant values are displayed in bold font.
